# *GADD45a* Promoter Regulation by a Functional Genetic Variant Associated with Acute Lung Injury

**DOI:** 10.1371/journal.pone.0100169

**Published:** 2014-06-18

**Authors:** Sumegha Mitra, Michael S. Wade, Xiaoguang Sun, Nurgul Moldobaeva, Carlos Flores, Shwu-Fan Ma, Wei Zhang, Joe G. N. Garcia, Jeffrey R. Jacobson

**Affiliations:** 1 Division of Pulmonary, Critical Care, Sleep and Allergy, Department of Medicine, University of Illinois at Chicago, Chicago, Illinois, United States of America; 2 Centro de Investigacion Biomedica en red Enfermedades Respiratorias, Instituto de Salud Carlos III, Madrid, Spain; 3 Research Unit, Hospital Universitario Nuestra Senora de Candelaria, Tenerife, Spain; 4 Division of Pulmonary, Critical Care and Sleep, University of Chicago, Chicago, Illinois, United States of America; 5 Department of Pediatrics, University of Illinois at Chicago, Chicago, Illinois, United States of America; 6 Arizona Health Sciences Center, University of Arizona, Tucson, Arizona, United States of America; University of Pittsburgh, United States of America

## Abstract

**Rationale:**

Growth arrest DNA damage inducible alpha (*GADD45a*) is a stress-induced gene we have shown to participate in the pathophysiology of ventilator-induced lung injury (VILI) via regulation of mechanical stress-induced Akt ubiquitination and phosphorylation. The regulation of *GADD45a* expression by mechanical stress and its relationship with acute lung injury (ALI) susceptibility and severity, however, remains unknown.

**Objectives:**

We examined mechanical stress-dependent regulatory elements (MSRE) in the *GADD45a* promoter and the contribution of promoter polymorphisms in *GADD45a* expression and ALI susceptibility.

**Methods and Results:**

Initial studies in *GADD45a* knockout and heterozygous mice confirmed the relationship of *GADD45a* gene dose to VILI severity. Human lung endothelial cells (EC) transfected with a luciferase vector containing the full length *GADD45a* promoter sequence (−771 to +223) demonstrated a >4 fold increase in *GADD45a* expression in response to 18% cyclic stretch (CS, 4 h) compared to static controls while specific promoter regions harboring CS-dependent MSRE were identified using vectors containing serial deletion constructs of the *GADD45a* promoter. *In silico* analyses of *GADD45a* promoter region (−371 to −133) revealed a potential binding site for specificity protein 1 (SP1), a finding supported by confirmed SP1 binding with the *GADD45a* promoter and by the significant attenuation of CS-dependent *GADD45a* promoter activity in response to SP1 silencing. Separately, case-control association studies revealed a significant association of a *GADD45a* promoter SNP at −589 (rs581000, G>C) with reduced ALI susceptibility. Subsequently, we found allelic variation of this SNP is associated with both differential GADD45a expression in mechanically stressed EC (18% CS, 4 h) and differential binding site of interferon regulatory factor 7 (IRF7) at this site.

**Conclusion:**

These results strongly support a functional role for *GADD45a* in ALI/VILI and identify a specific gene variant that confers risk for ALI.

## Introduction

Acute lung injury (ALI) and its more severe form, acute respiratory distress syndrome, are complex disorders that are precipitated by the interplay of both environmental factors (such as mechanical ventilation) and genetic factors. Several case-control association studies have identified specific single nucleotide polymorphisms (SNPs) that contribute to ALI susceptibility and survival [1]–[3]. In this regard, we have previously employed preclinical models of ALI and global gene expression profiling to identify several ALI candidate genes, including *GADD45a,* and ALI-associated SNPs [4]–[8]. As these studies have yielded important insights into ALI pathobiology and implicated specific genetic variants associated with ALI risk and severity, further research may ultimately lead to novel therapeutic targets that bring personalized medicine to the fore in strategies aimed at treating or preventing ALI.

Growth arrest DNA damage inducible alpha (*GADD45a*) is a stress-induced gene which we previously reported to be significantly upregulated in multi-species pre-clinical models of ventilator-induced lung injury (VILI) [9]. We subsequently reported that mice lacking *GADD45a* gene (*GADD45a* ^−/−^) demonstrated significantly increased VILI susceptibility [10] and linked this observation to effects of GADD45a depletion on the differential ubiquitination of Akt resulting in both increased proteasomal degradation of Akt and decreased Akt phosphorylation in response to mechanical stress [11]. However, the regulation of *GADD45a* expression in response to mechanical stress and the association of *GADD45a* genetic variants with ALI/VILI susceptibility are largely unknown.

In the present study, we hypothesized the existence of *GADD45a* SNPs that are associated with functional effects on promoter activity and GADD45a expression levels as well as ALI susceptibility. We relied on complementary approaches including the use of *GADD45a* promoter deletion constructs in endothelial cells (EC) subjected to cyclic stretch (CS) to determine regions harboring mechanical stress response elements (MSRE) followed by ALI case-control association studies focused on specific promoter regions of interest to identify *GADD45a* SNPs that are associated with both functional effects on GADD45a promoter activity in response to mechanical stress and ALI clinically. Our results provide evidence for ALI/VILI susceptibility conferred by specific *GADD45a* genetic variants that further supports an important role for *GADD45a* in susceptibility to inflammatory lung injury.

## Materials and Methods

### EC Culture and *in vitro* Cyclic Stretch

Human pulmonary artery endothelial cells (EC) (Lonza, US-Allendale, NJ) were plated onto BioFlex silicone elastomer six-well plates coated with type I collagen and were cultured in endothelial growth medium (EGM-2) containing 10% FBS (Lonza, US-Allendale, NJ) in 5% CO_2_ at 37°C and 95% humidity to achieve contact-inhibited monolayers. For mechanical stress studies, BioFlex plates were placed on a Flexcell Strain Tension System (FX-3000, Flexcell International, Hillsborough, NC) kept in a 5% CO_2_ incubator at 37°C and 95% humidity. Plates were stretched to produce either 5% or 18% elongation at a frequency of 0.5 Hz, 30 cycles/min. As we have previously reported, 18% cyclic stretch (CS) corresponds to pathologically relevant levels of mechanical stress that result in phenotypic EC monolayer changes, increased susceptibility to barrier-disruptive agonists, but with preserved monolayer integrity even after prolonged exposure (48 h) [12].

### *GADD45a* Promoter Vector and Molecular Cloning

*GADD45a* promoter cloned into pSGG luciferase vector was purchased from SwitchGear Genomics (S119097, Menlo Park, CA). *In silico* analysis and gene sequencing of the vector confirmed 1008 bp insert (−771 to +237) spanning regions of the *GADD45a* promoter and exon 1, 80 bp away from the transcription start site. Primers were then designed at every 200 bp of the *GADD45a* promoter sequence (Table S1) and amplified PCR fragments of 806, 606, 368, and 172 bp sizes were cloned into an empty pSGG luciferase vector to generate 5′ serial deletion constructs of *GADD45a* promoter. Cloning results were confirmed by DNA sequencing using primers (forward: TCCATCAAAACAAAACGAAACAA and reverse: CCGTCTTCGAGTGGGTAGAATG) sequences provided by SwitchGear Genomics (Menlo Park, CA).

### Dual Luciferase Reporter Gene Assay

EC were co-transfected with *GADD45a* plasmid constructs containing firefly luciferase reporter (1 µg) and TK renilla vector (20 ng) using Fugene HD transfection reagent (Promega, Madison, WI, USA). Dual luciferase activity was measured using Luciferase Assay reagent II and Stop & Glo reagent (Promega, Madison, WI) according to the manufacturer’s protocol. Normalized luciferase activity was expressed as ratio of firefly and renilla luciferases activities.

### Site-directed Mutagenesis

A point mutation was created in the *GADD45a* promoter sequence (at −589) to create promoter SNP rs581000 (G>C) using primer sequences (5′-acaaacgggttggtttttctttttt*c*agcttccaaccct-3′ and 5′-agggttggaagct*g*aaaaaagaaaaaccaacccgtttgt-3′) obtained by quick change primer design software and using QuikChange Lightning Site-Directed Mutagenesis Kit (Agilent Technologies, Santa Lara, CA) according to manufacturer’s protocol. Mutagenesis was verified by DNA sequencing.

### In silico Analysis

The core and matrix similarity of TF binding sites in the *GADD45a* promoter region were evaluated using Genomatix software (http://www.genomatix.de).

### Electrophoretic Mobility Shift Assay (EMSA)

EMSA was performed using a LightShift Chemiluminescent EMSA Kit (Thermo Scientific, Asheville, NC, USA) and biotin-labeled synthetic oligonucleotides carried either the G or C allele of the SNP rs581000 (−589) or the *GADD45a* promoter region (−191 to −140) with SP1 binding sites. Non-biotin-labeled synthetic oligonucleotides with the consensus sequences for IRF7 and SP1 were used as competitors. Nuclear cells extracts from HeLa and HL60 cells (Promega, Madison, WI) were incubated with biotin-labeled oligonucleotides (20 fmol) in presence of IRF7 and SP1 competitor respectively and eletrophoresed on 6% Novex DNA retardation gels (Invitrogen, Carlsbad, CA, USA) as described elsewhere [13]. The images were obtained by chemiluminescence according to the manufacturer’s instructions.

### Transfection of siRNA

EC were transfected with SP1 siRNA (100 nM, Dharmacon Thermo Scientific, Pittsburgh, PA) or non-specific, scrambled sequence RNA using transfection reagent siPORT *Amine* (Ambion, Austin, TX, USA) in serum-free conditions according to the manufacturer’s protocol. siRNA and plasmid co-transfection was performed using Lipojet (SignaGen Laboratories, Rockville, MD) according to the manufacturer’s protocol.

### Western Blotting

Total proteins were extracted using NP-40 lysis buffer (50 mM TrisHCl pH 7.4, 150 mM NaCl, 1% NP-40, and 5 mM ethylenediaminetetraacetic acid) supplemented with 40 mM sodium fluoride, 0.1 M sodium orthovanadate, 0.2 mM phenylmethylsulfonyl fluoride, 10 mM N’ ethyl malamide, and protease and phosphatase inhibitor cocktail (Calbiochem, San Diego, CA). Protein concentration was measured using bicinchoninic acid (BCA) protein assay kit (Pierce, Rockford, IL) and Western blotting was performed using standard protocols using SP1 antibody (Cell Signaling, Danvers, MA).

### Study Populations and Demographics

The association of *GADD45a* SNPs with ALI was studied in case-control samples from Chicago, IL comprised of African American (AA) and European American (EA) subjects. A detailed description of the population studied is provided in Table 1 and in our previous report [5]. A total of 208 unrelated severe sepsis cases (137 EA and 71 AA) including 114 with ALI (74 EA and 40 AA) and 368 healthy population-based controls subjects (186 EA and 182 AA) were collected. Control subjects from this group reported a negative personal and first-degree family history of sepsis or ALI. Ancestry was defined as at least three grandparents of either AA or EA ancestry. Severe sepsis and ALI were defined according to the American-European Consensus Criteria [14] and the Society of Critical Care Medicine consensus statement [15]. Additionally, samples from the Canary Islands, Spain that included 95 population-based controls and 80 severe sepsis cases including 66 with ALI were also studied [6]. Spanish cases were admitted to an ICU within 24 h of a diagnosis of severe sepsis. Further details about regarding each study population is available in Table 1. To account for population stratification, the association study was performed in AA, EA and Spanish individuals separately. A total of 93, 96 and 20 ancestry informative markers were previously genotyped by our group in EA, AA and Spanish subjects, respectively, which suggested limited influence of population stratification on association results within each study sample [5], [6]. The study was approved by the University of Illinois Institutional Review Board and was in accordance with Helsinki rules.

**Table 1:** Population characteristics of Chicago and Spanish samples studied.

Characteristics	Sepsis+ALI			ALI		
	AA	EA	Spanish	AA	EA	Spanish
**Sample Size**	71	137	80	40	74	66
**Gender ^#^**	31/40	71/66	58/22	19/21	41/33	54/12
**Age** *	54.9±19.3	61.2±15.8	65.2±12.3	54.6±15.3	57.7±16.9	58.4±12.2
**APACHE II** *	27.1±7.0	28.4±7.6	22.4±5.8	28.0±7.7	29.7±7.4	20.1±5.4
**Survival %**	62.7	64.1	56.1	52.4	62.1	52.8
**Smoking (%)**	37.7	38.8	30.4	34.2	40.3	29.5
**Diabetes (%)**	20.0	19.7	21.4	16.7	19.0	18.6
**Renal Failure (%)**	29.3	19.0	–	21.4	19.0	–
**Characteristics**	**Controls**					
	**AA**	**EA**	**Spanish**			
**Sample Size**	182	186	95			
**Gender ^#^**	90/92	84/102	53/42			
**Age** *	56.6±17.4	55.6±14.4	48.9±12.3			
**APACHE II** *	NA	NA	NA			
**Survival %**	NA	NA	NA			
**Smoking (%)**	–	–	30.5			
**Diabetes (%)**	0.0	0.0	9.5			
**Renal Failure (%)**	0.0	0.0	–			

*expressed as mean ± SD.

APACHE II: Acute Physiology and Chronic Health Evaluation.

# Male/Female.

- Data not available.

### Human *GADD45a* Gene Resequencing

DNA samples of 30 randomly selected ALI cases (15 AA and 15 EA) from the Chicago study were sequenced for *GADD45a* SNP discovery. DNA sequencing and polymorphism identification were performed by Polymorphic DNA Technologies (Alameda, CA) according to established guidelines. The genomic sequence NM_001924.3 was used as the reference sequence.

### Genotyping

Genotyping was conducted using the iPLEX Gold Platform (Sequenom, San Diego, CA) according to the manufacturer’s protocol. Briefly, iPLEX assays were scanned by matrix-assisted laser desorption/ionization time-of-flight mass spectrometry and individual SNP genotype calls were automatically generated with Sequenom TYPER 3.4 software. Genotyping was validated by the TaqMan allelic discrimination assay (Life Technologies, Carlsbad, CA). TaqMan genotyping was performed using a 7900HT Fast Real-Time PCR System (Life Technologies, Carlsbad, CA), with automated calls generated by SDS software based on discriminating plots (95% confidence). Genotyping was blind to case-control status and ethnic background of the samples.

### Preclinical Model of VILI

Male 8 to 12 week-old wildtype (WT) C57BL/6 (Jackson Laboratory, Bar Harbor, ME), *GADD45a^−/−^* (a gift of Dr. Michael O’Reilly, University of Rochester and Dr. Albert Fornace, Brigham and Women’s Hospital) and *GADD45a* ^+/−^ (*GADD45a* ^−/−^ crossed with C57BL/6) were subjected to mechanical ventilation (Harvard Apparatus, Holliston, MA) with tidal volumes of 40 ml/kg, 65 breaths per minute for 4 h as we have previously described [11]. All the *GADD45a* ^−/−^ and *GADD45a* ^+/−^ mice were genotyped by PCR as described elsewhere [16]. All animal experiments were approved by the Animal Care and Use Committees of the University of Illinois at Chicago.

### Statistical Analysis

Individual SNP association testing with ALI and odds ratio estimates with 95% confidence intervals (CI) was performed using SNPstats software assuming two genetic models, additive and dominant [17]. To account for the multiple-testing, raw p values from tests in each cohort were adjusted using the Benjamini-Horchberg procedure [18]. Departures from Hardy-Weinberg equilibrium were tested by means of exact test [19]. Haploview software was used to study the correlation and linkage disequilibrium (LD) between SNPs [20]. The multiple-marker selection algorithm haplotype r2 included in TagIT 3.03 software was used to select a set of SNPs (tSNPs) [21]. Student t-test was used for comparison of promoter activity and protein expression between different groups. Statistical significance was set at a p value of less than 0.05.

## Results

### *GADD45a* Expression Levels and Susceptibility to Ventilator-induced Lung Injury (VILI)

**Figure 1:**
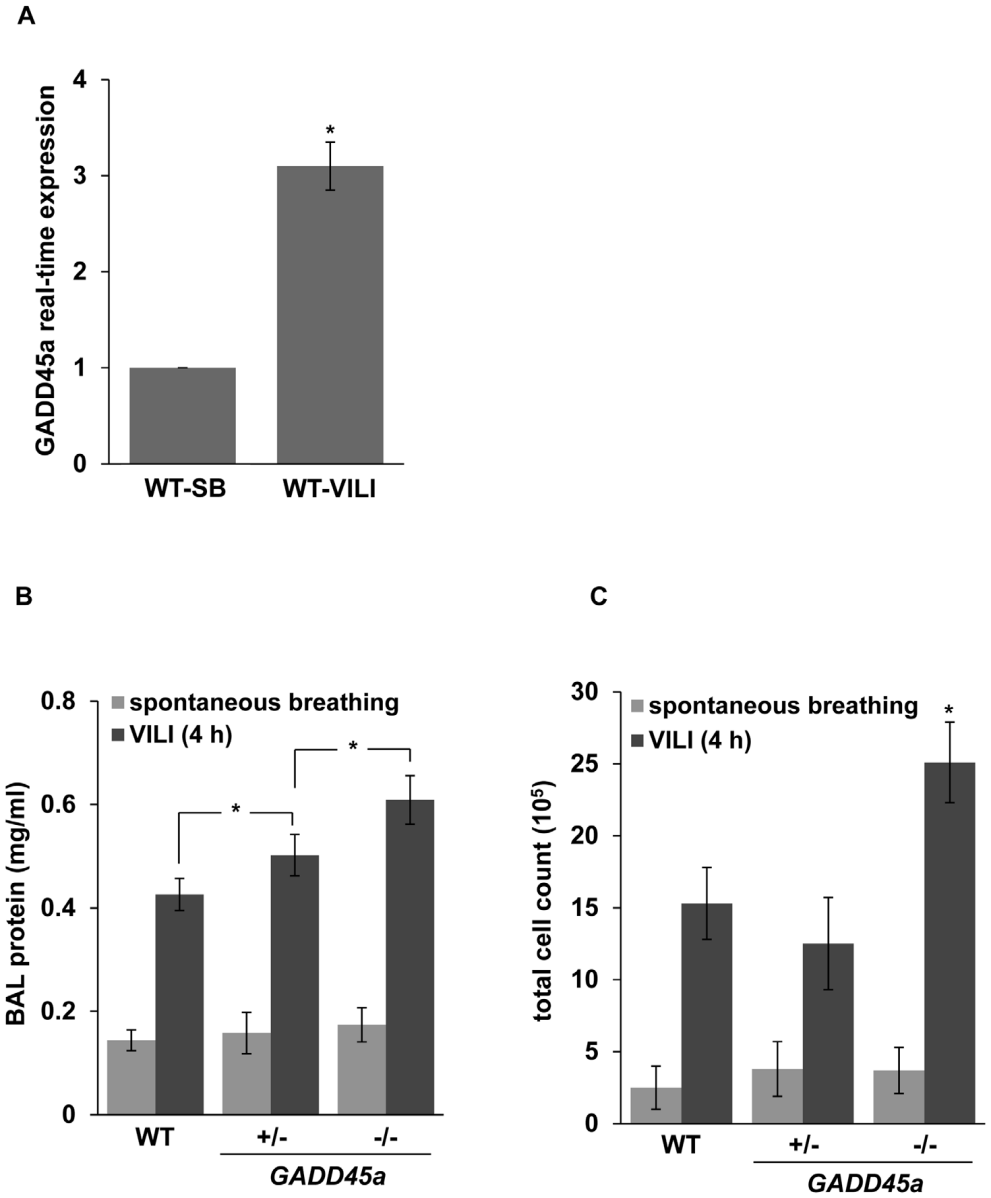
GADD45a expression and mechanical stress-induced murine lung injury. (**A**) *GADD45a* mRNA levels from lung homogenates of wildtype (WT) mice subjected to high tidal volume mechanical ventilation (V_T_ = 40 ml/kg, 4 h) were significantly increased in comparison to spontaneously breathing (SB) control mice (n = 3/group, *p<0.05). (**B**) WT, *GADD45a* heterozygous (*GADD45a* ^+/−^), and *GADD45a* knockout (*GADD45a^−/−^*) mice were subjected to high tidal volume mechanical ventilation (V_T_ 40 ml/kg, 4 h) and BAL fluid was collected for analyses. BAL fluid total protein levels were significantly higher in *GADD45a* ^+/−^ mice compared to WT and significantly less than *GADD45a^−/−^* mice. SB animals showed no difference (n = 3/group, *p<0.05). (**C**) Total cell counts in BAL fluid from VILI-challenged *GADD45a^−/−^* mice were significantly increased compared to both *GADD45a^+/−^* and WT animals while there was no difference was observed between WT and *GADD45a* ^+/−^ animals after VILI challenge (n = 3/group, *p<0.05).

We previously identified *GADD45a* as a significantly upregulated gene in multi-species pre-clinical models of VILI and reported increased VILI susceptibility in *GADD45a* knockout (*GADD45a* ^−/−^) mice [10], [11]. To extend these earlier studies, we initially assessed GADD45a mRNA levels by real-time PCR in spontaneously breathing (SB) and VILI-challenged (V_T_ 40 ml/kg, 4 h) wildtype (WT) mice and confirmed significantly increased GADD45a mRNA levels in response to mechanical stress (Figure 1A). Next, to confirm that variable *GADD45a* expression levels are associated with variable susceptibility and severity of ALI *in vivo*, WT, *GADD45a* heterozygous mice (*GADD45a* ^+/−^) and *GADD45a* ^−/−^ mice were subjected to VILI challenge (V_T_ 40 ml/kg, 4 h) and BAL fluid was collected for analysis. In response to VILI challenge, these studies confirmed that heterozygous *GADD45a* ^+/−^ mice exhibit an intermediate phenotype with significantly increased BAL protein levels compared to WT mice and but significantly decreased BAL protein levels compared to *GADD45a* ^−/−^ mice (Figure 1B). BAL total cell counts were notable for a significant increase in VILI-challenged *GADD45a* ^−/−^ mice compared to both WT and *GADD45a* ^+/−^ animals while there were no significant differences noted between WT and *GADD45a* ^+/−^ animals (Figure 1C). No changes were observed between groups of spontaneously breathing animals. These results support a critical role for *GADD45a* expression in murine VILI response.

### *GADD45a* Promoter Activity in Response to Mechanical Stress

**Figure 2:**
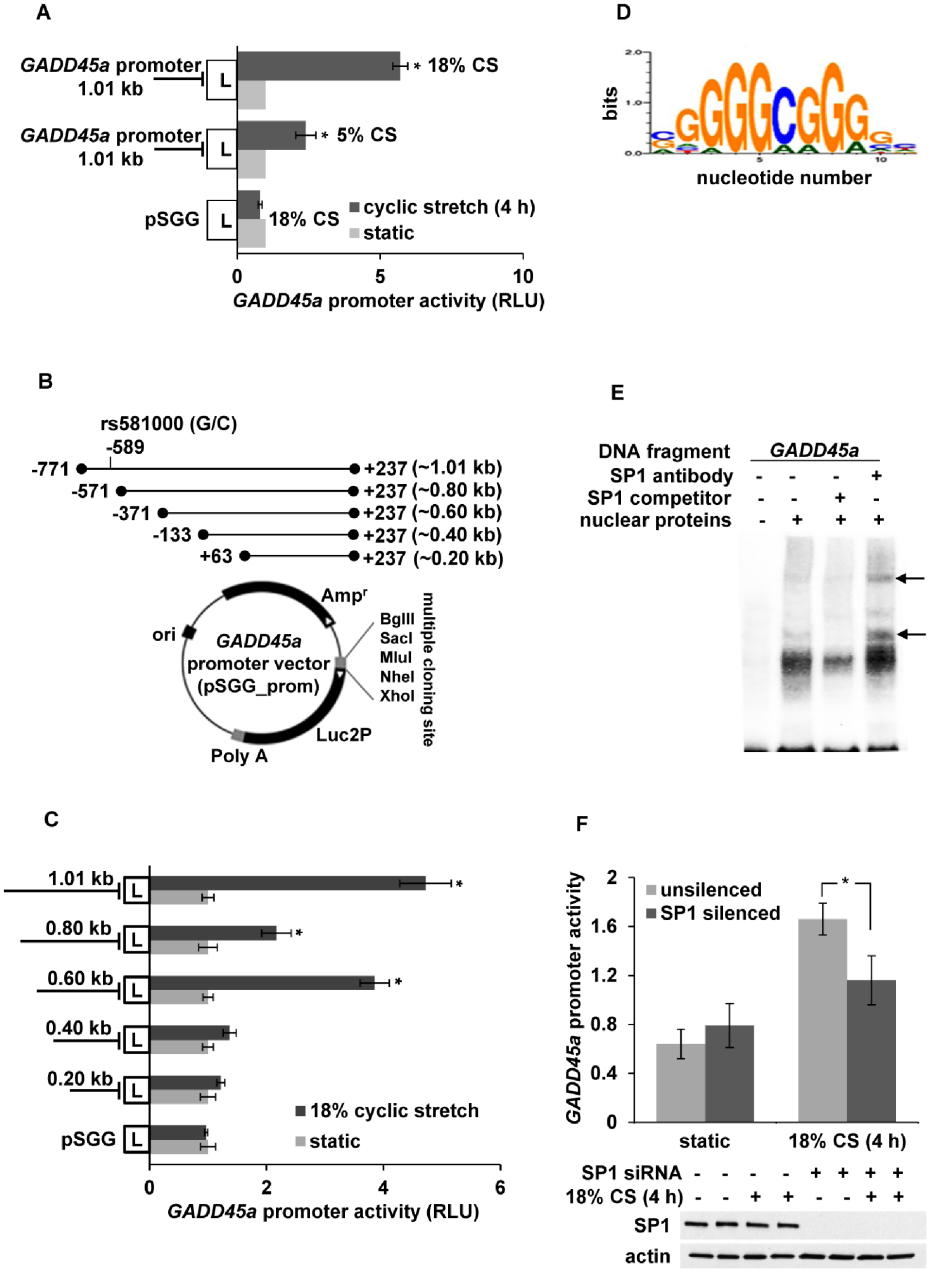
*GADD45a* promoter activity and functional promoter region in response to mechanical stress. (**A**) Human pulmonary artery endothelial cells (EC) were plated on Bioflex stretch plates and transfected with a full-length *GADD45a* promoter vector followed by cyclic stretch (CS, 5% or 18%) for 4h. Dual luciferase reporter assay revealed about significant increases in *GADD45a* promoter activity proportional to the degree of CS (n = 3/condition, * p<0.05 compared to respective static controls). (**B**) Schematic representation of full-length and deletion fragments of *GADD45a* promoter and empty pSGG reporter vector. Promoter fragments with 200 bp serial deletions were PCR amplified and cloned into empty pSGG vector to generate *GADD45a* promoter constructs. (**C**) EC were transfected with *GADD45a* promoter deletion constructs followed by 18% CS (4 h). Dual luciferase reporter assay revealed ∼4 fold increase in reporter activity in both full-length (1.01 kb) and 0.6 kb (−371 to +237) *GADD45a* promoter fragment in response to 18% CS cells compared to respective static controls. Comparatively, 18% CS-induced luciferase activity was significantly decreased in 0.8 kb, 0.4 kb and 0.2 kb fragments. (**D**) *In silico* analysis of *GADD45a* promoter region −371 to −133 by Genomatix predicted binding sites for transcription factor SP1. The sequence logo graphically represents the SP1 consensus sequence. The relative height of each base within each stack represents relative frequency of the corresponding base at that position. Highly conserved positions are represented by higher stacks of base symbols A, T, G, C. (**E**) The binding of SP1 with the *GADD45a* promoter was detected by EMSA using biotin-labeled *GADD45a* promoter fragment and HL60 nuclear extract in the presence or absence of a non-labeled SP1 competitor and antibodies specific for SP1. DNA-protein interaction was characterized by complex formation upon the addition of nuclear proteins, which was blocked in the presence of an SP1 competitor. Addition of SP1 antibody altered the mobility of the complex, characterized by a super-shift of DNA-protein complex (arrow). (**F**) EC co-transfected with SP1 siRNA and a full-length *GADD45a* promoter vector followed by 18% CS (4 h) exhibited significantly attenuated *GADD45a* promoter activity compared to unsilenced cells transfected with the full-length *GADD45a* promoter vector alone (n = 3/condition, * p<0.05). Silencing was confirmed by Western blotting (representative blots shown).

To assess the effect of mechanical stress on human *GADD45a* promoter activity, we exposed human pulmonary artery endothelial cells (EC) transfected with pSGG luciferase vector containing a human *GADD45a* promoter sequence (−771 to +237, 1.01 kb) to either 5% or 18% cyclic stretch (CS, 4h). Compared to static controls, 5% and 18% CS induced ∼2.5- and ∼6-fold increases in luciferase activity, respectively, confirming *GADD45a* promoter regulation by mechanical stress (Figure 2A).

To identify the potential *GADD45a* promoter regions harboring mechanical stress response elements, EC were transfected with either a full length *GADD45a* promoter vector or one of the four promoter deletion vectors (Figure 2B) prior to exposure to excessive mechanical stress *(*18% CS, 4 h). These studies revealed no significant increase in mechanical stress-induced *GADD45a* promoter activity with the fragments representing the proximal promoter nucelotides +63 to +237 (0.2 kb) or −133 to +237 (0.4 kb) (Figure 2C). However, luciferase activity was significantly increased with the promoter fragment from −371 to +237 (0.6 kb), indicating the presence of MSRE in the region of nucelotides −371 to −133. However, further extension distally to include nucleotides −571 to +237 (0.8 kb) was associated with a significant decrease in promoter activity in response to CS indicating elements that repress transcription in this region. Notably, the full length promoter (−771 to +237; 1.01 kb) overrode these effects and restored mechanical stress-dependent *GADD45a* promoter activity (Figure 2C), suggesting presence of *cis*-regulatory elements in this region (−771 to −571). No effects were observed with any of the vectors under static conditions.

Next, using Genomatix software we performed the *in silico* analysis of the *GADD45a* promoter region from −371 to −133, which predicted binding sites (Figure 2D) for specificity protein 1 (SP1), a transcription factor ubiquitously expressed in all mammalian cells [22], at positions −232, −202, −177 of the *GADD45a* promoter. To validate this finding, we performed EMSA with nuclear extracts from HL60 cells (human promyelocytic leukemia cells) in the presence or absence of a non-biotin-labeled SP1 competitor and antibodies specific for SP1. These experiments identified DNA-protein interaction by the complex formed between the biotin-labeled *GADD45a* promoter fragment (range: −191 to −140 and length: 51mer) and HL60 nuclear proteins that was abrogated in the presence of the SP1 competitor (Figure 2E). To confirm the presence of SP1 in the DNA-protein complex, antibodies against SP1 were added to the binding reaction prior to adding the probe at 4°C. A significant shift in the DNA-protein complex was observed consistent with an alteration in the mobility of the complex due to binding of SP1 antibody with the DNA-protein complex (Figure 2E). Moreover, silencing SP1 in EC transfected with the full-length *GADD45a* promoter vector significantly reduced 18% CS-induced *GADD45a* promoter activity compared to unsilenced control cells (Figure 2F). Taken together, these studies indicate SP1 is an important regulator of EC GADD45a expression induced by excessive mechanical stress.

### *GADD45a* Genetic Variants and Association of Promoter SNP rs581000 with Acute Lung Injury (ALI)

Resequencing of the *GADD45a* gene and 2 kb of upstream and downstream regulatory elements identified 31 variations (29 SNPs and 2 indels) including two variants not previously identified (Table 2). Seven *GADD45a* tagging SNPs (tSNPs) with minor allele frequency (MAF) >0.10 were genotyped in both Chicago and Spanish samples (Table 2). A case–control association study was performed in the Chicago cohort, comprised of 208 severe sepsis cases including 114 with ALI (137 EA and 71 AA) and 368 healthy controls (186 EA and 182 AA), and in the Spanish cohort comprised of 80 severe sepsis cases, 66 with ALI, and 95 healthy controls. The population characteristics of the cohorts studied is provided in Table 1. The assessment of ancestry informative markers suggested no significant difference in the genetic background of cases and controls in these cohorts [5], [6]. Additionally, to account for population stratification, the association study was performed separately in AA, EA and Spanish individuals.

**Table 2:** Summary of SNPs in *GADD45a* gene.

SNP ID	Position^a^	Location^b^	Allele	Frequ ency AA^c^	Frequency EA^c^	Tagging SNP
rs1511686	68,148,921	c.-318-1939	T>A	0.13	0.00	
–	68,149,189	c.-318-1671	G>A	0.00	0.03	
rs115517134	68,149,321	c.-318-1539	A>G	0.03	0.00	
rs188178283	68,150,258	c.-318-602	G>T	0.00	0.03	
**rs581000**	**68,150,271**	**c.-318-589**	**G>C**	**0.37**	**0.13**	**
rs3783456	68,150,622	c.-318-238	A>G	0.03	0.00	
rs3783457	68,150,673	c.-318-187	C>A	0.03	0.00	
–	68,150,681	c.-318-179	C>G	0.03	0.00	
**rs1397946**	**68,150,898**	**c.-280**	**A>T**	**0.13**	**0.00**	**
rs3783460	68,150,931	c.-247	T>G	0.03	0.00	
rs3783462	68,151,227	c.44+6	T>A	0.03	0.00	
rs3783465	68,151,645	c.45-63	G>A	0.03	0.00	
rs3783466	68,151,685	c.45-23	C>T	0.17	0.13	
rs2759219	68,151,980	c.147-53	G>T	0.3	0.13	**
rs149179509	68,152,171	c.285	G>A	0.00	0.03	
**rs3783468**	**68,152,363**	**c.384+93**	**G>A**	**0.23**	**0.57**	**
rs3783469	68,152,386	c.384+116	T>C	0.03	0.13	**
rs681673	68,152,388	c.384+118	C>T	0.5	0.27	
rs532446	68,152,438	c.384+168	T>C	0.5	0.27	**
rs3783472	68,152,558	c.384+288	G>C	0.03	0.00	
rs143275022	68,152,736	c.384+466	del T	0.00	0.03	
**rs3171012**	**68,153,206**	**c.385-138**	**T>C**	**0.17**	**0.13**	**
rs3046000	68,153,271–68,153,279	c.385-73_385-65	del (9nt)	0.1	0.00	**
**rs3783478^#^**	**68,153,451**	**c.492**	**A>G**	**0.13**	**0.00**	**
rs3783479	68,153,484	c.*27	C>T	0.03	0.00	
**rs607375**	**68,154,086**	**c.** * **564+65**	**C>G**	**0.4**	**0.27**	**
rs3783482	68,154,204	c.*564+183	C>T	0.03	0.00	
rs3783483	68,154,208	c.*564+187	G>A	0.07	0.00	**
**rs606470**	**68,154,299**	**c.** * **564+278**	**T>C**	**0.23**	**0.03**	**
rs77985416	68,154,373	c.*564+352	G>A	0.03	0.00	
Rs78379425	68,154,466	c.*564+445	G>A	0.03	0.00	

a Position on chromosome 1 according to NCBI Build 37.

b Location relative to reference sequence NM_001924.3.

c Represents minor allele frequency in gene resequencing data in 15 African American (AA) and 15 European American (EA).

Boldface highlights SNPs genotyped in both Chicago and Spanish study.

** Represents tagging SNP.

* Represents 3′UTR region.

# represents synonymous SNP (Glu164Glu).

**Figure 3:**
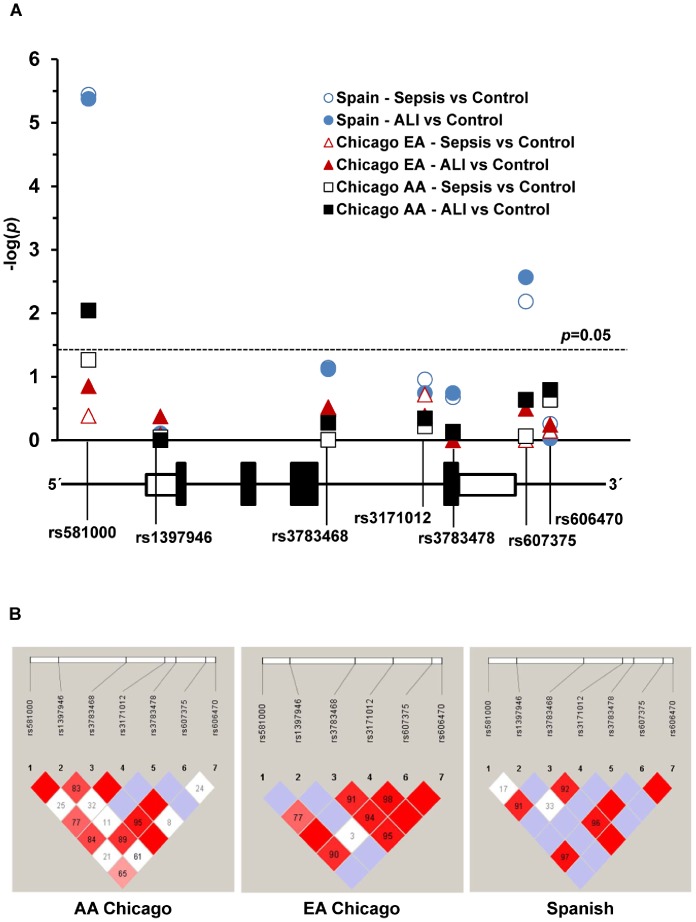
*GADD45a* SNP association with severe sepsis and ALI. (**A**) Plots of association *P* values of tested *GADD45a* SNPs identified significant association of promoter SNP rs581000 with ALI in AA of Chicago cohort and with both severe sepsis and ALI in Spanish cohort. In Spanish cohort intergenic SNP rs607375 was associated with both severe sepsis and ALI. The dashed line represents a *p*-value of 0.05 and a schematic of the *Gadd45a* gene structure below indicates relative position of SNPs tested for association. Black boxes within the gene schematic represent coding exons. White boxes represent the 5′ and 3′ UTR, respectively. (**B**) Panel represents linkage disequilibrium (LD) patterns across the genotyped SNPs in the two cohorts. High LD is noted between promoter SNP rs581000 and rs607375 in both EAs from the Chicago cohort and in the Spanish cohort. Each diamond of the LD plot represents a pairwise SNP comparison. Numbers and colors in each diamond indicate the magnitude of LD between pairs of SNPs (D′ = 100 corresponds to complete LD denoted by red; D′ = 0 corresponds to absence of LD denoted by white; blue represents an intermediate association).

These studies identified a significant association of the *GADD45a* promoter SNP rs581000 with allele C reducing the risk of both sepsis and ALI in AAs from Chicago (p = 0.009, adjusted p = 0.05 under a dominant model) and in Spanish subjects (p = 4.20E-06, adjusted p value = 0.00003 under a dominant model, FDR <1%) (Figure 3A and Table 3). However, in EA from Chicago, the association did not reach to a significant level. Additionally, none of the other tSNPs resulted in significant association with sepsis/ALI in EAs or AAs subjects from Chicago. In Spanish subjects, reduced sepsis/ALI susceptibility was also significantly associated with the intergenic SNP rs607375 (p = 0.0027, adjusted p = 0.009 under a dominant model, FDR <1%) (Figure 3A). Moreover, the promoter SNP rs581000 demonstrated nearly complete LD with the intergenic SNP rs607375 in the Spanish cohort (LD; D′ = 0.97) and EAs from the Chicago cohort (LD; D′ = 0.90) (Figure 3B). In AAs, the promoter SNP rs581000 was in complete LD with promoter SNP rs1397946 (D′ = 100) and in partial LD with a synonymous exon SNP rs3783478 (D′ = 84). The Hardy-Weinberg equilibrium was observed in nearly all the comparisons, although an exception was noted for rs581000 in Spanish controls. Notably, the MAF of rs581000 in our Spanish controls (0.28) was comparable to that in a Hispanic reference panel (0.24) from dbSNP [23]. These data strongly implicate an association between *GADD45a* rs581000 and sepsis/ALI susceptibility.

**Table 3:** *GADD45a* promoter SNP rs581000 genotypes in Chicago and Spanish cohorts.

Chicago study	GG	GC	CC	Frequency (GC+CC)	Odds Ratio (CI)	p value
Controls (n = 368)	215	134	19	0.42		
Severe sepsis (n = 208)	139	59	10	0.33	0.70 (0.49–0.95)	0.05
Sepsis+ALI (n = 114)	76	31	7	0.33	0.70 (0.45–1.09)	0.12
**AA Chicago study**	**GG**	**GC**	**CC**	**Frequency (GC+CC)**	**Odds Ratio (CI)**	**p value**
Controls (n = 182)	68	99	15	0.63		
Severe sepsis (n = 71)	36	28	7	0.49	0.58 (0.33–1.01)	0.05
Sepsis+ALI (n = 40)	24	12	4	0.40	0.40 (0.20–0.80)	0.009
**EA Chicago study**	**GG**	**GC**	**CC**	**Frequency (GC+CC)**	**Odds Ratio (CI)**	**p value**
Controls (n = 186)	147	35	4	0.21		
Severe sepsis (n = 137)	103	31	3	0.25	1.24 (0.74–2.10)	0.41
Sepsis+ALI (n = 74)	52	19	3	0.30	1.59 (0.87–2.94)	0.14
**Spanish study**	**GG**	**GC**	**CC**	**Frequency (GC+CC)**	**Odds Ratio (CI)**	**p value**
Controls (n = 95)	44	50	1	0.54		
Severe sepsis (n = 80)	64	14	2	0.20	0.22 (0.11–0.43)	5x10^−6^
Sepsis+ALI (n = 66)	54	10	2	0.18	0.19 (0.09–0.40)	6x10^−6^

AA represents African American and EA represents European American in the Chicago study.

### Functional Assessment of *GADD45a* Promoter SNP rs581000

**Figure 4:**
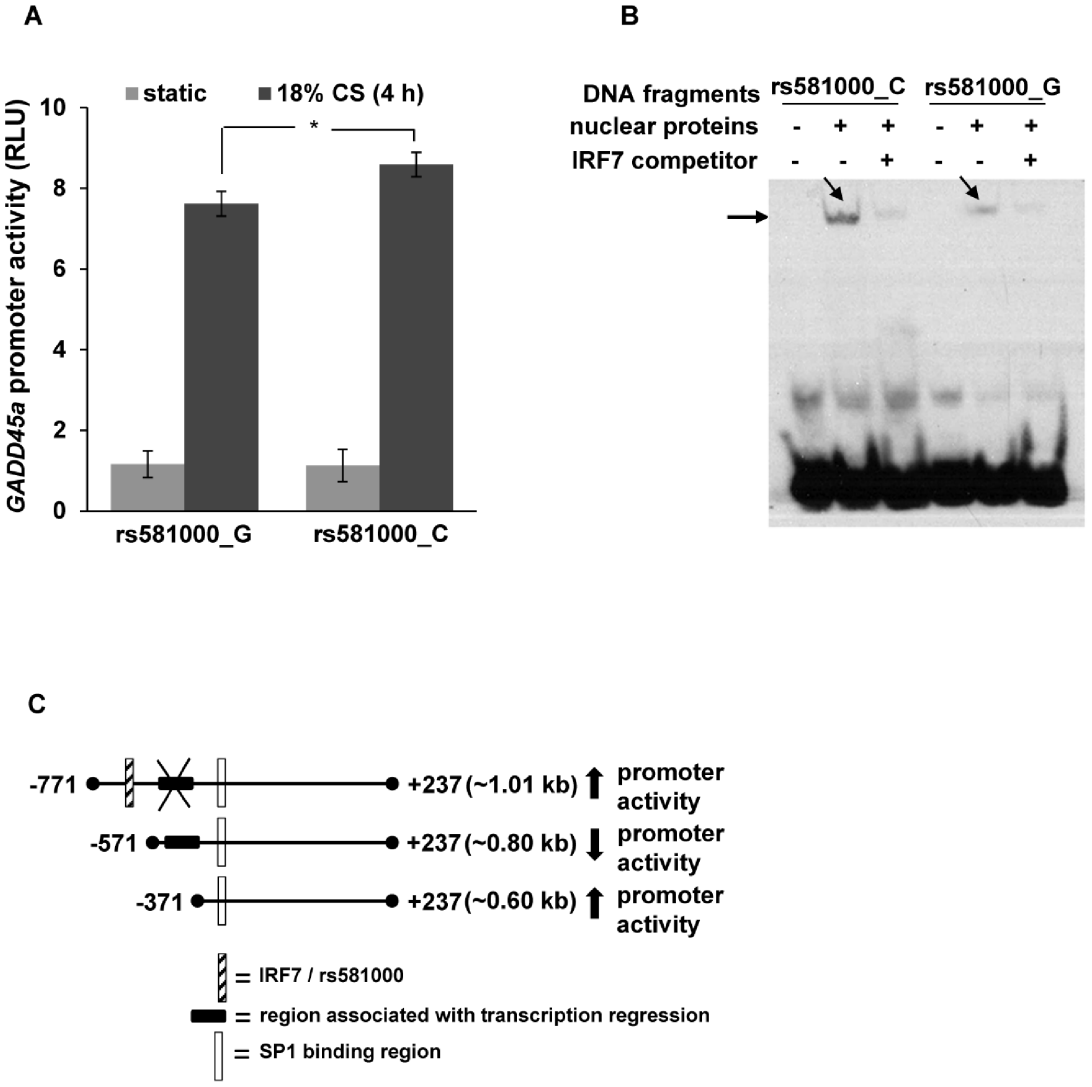
Promoter SNP rs581000 effect on mechanical stress-induced *GADD45a* activity. (**A**) EC were transfected with rs581000_C or rs581000_G vectors and then subjected to 18% CS (4 h). Cells transfected with rs581000_C vector demonstrated significantly increased luciferase reporter activity in response to CS compared to rs581000_G. (**B**) EMSA was performed with nuclear extracts from HeLa cells and biotin-labeled *GADD45a* promoter fragments with either the C allele or G allele in the presence or absence of an interferon regulatory factor 7 (IRF7) competitor. Binding affinity of the allelic variants with nuclear proteins is indicated by arrows (arrows = protein-bound DNA). (**C**) Schematic representation of *GADD45a* promoter regions with regulatory elements. SNP rs581000 creating c*is*-regulatory element IRF7 binding site, in the region −771 to −571 are associated with enhancement of promoter activity that overrides putative transcription suppressors in the region −571 to −371 and contributes to regulatory element in the region −371 to −133. Consistent with the deletion of the region −771 to −571 results in significant reduction of promoter activity but is restored by further deletion of region −571 to −371.

To determine the functional role of the *GADD45a* promoter SNP rs581000 (G>C), we used site-directed mutagenesis to create a point mutation at position −589 of the full-length (1.01 kb) *GADD45a* promoter fragment. Luciferase reporter activity was evaluated in EC expressing either the *GADD45a* vector allele rs581000_C or rs581000_G under static condition and in response to mechanical stress (18% CS, 4h). EC expressing the C allele at −589 (rs581000_C) exhibited significantly increased reporter activity after CS compared to cells expressing the G allele at the same locus (rs581000_G) (Figure 4A). These findings suggest that this allelic variant may correspond to alterations at a specific transcription factor-binding site resulting in variable transcription factor binding and gene expression. *In silico* analysis using Genomatix predicted that the substitution of G by C at the rs581000 locus results in gain of core sequence for the transcription factor, interferon regulatory factor 7 (IRF7). To investigate the potential contribution of IRF7 to *GADD45a* expression induced by mechanical stress, we performed EMSA with nuclear extracts from HeLa cells and biotin-labeled oligonucleotide containing the C or G allele (range: −614 to −561) in the presence or absence of competitive non-biotin-labeled oligonucleotides for IRF7. These studies revealed a 3.8 times stronger protein-DNA interaction with allele rs581000_C compared to allele rs581000_G (Figure 4B). This binding affinity of allele rs581000_C was abrogated in the presence of IRF7 competitive elements (Figure 4B) suggesting a significant role for SNP rs581000 in transcriptional regulation of *GADD45a* via altered binding affinity for IRF7. Collectively, these data support the idea that variable *GADD45a* expression by excessive mechanical stress is influenced by the cumulative effects of the promoter SNP rs581000, via alterations in DNA binding of IRF7, with overriding effects on an inhibitory region (−571 to −371) and by the additive effect of IRF7 and SP1 (Figure 4C).

## Discussion

VILI represents a form of ALI that is precipitated by excessive lung stretch generated by mechanical ventilation. Despite advances in ventilator strategies aimed at reducing the incidence of VILI through the administration of low tidal volumes [24], inflammation, pulmonary edema, and underlying lung disease may yet all contribute to poor compliance that result in an increased risk of injury associated with mechanical ventilation. As ALI remains a common and potentially devastating syndrome with mortality in excess of 35% [25], a better understanding of the underlying pathogenic mechanisms that may lead to the identification of novel therapeutic targets and molecular markers are desperately needed. Along these lines, mounting evidence suggests that ALI/VILI susceptibility and severity are influenced by a complex interplay involving genes and environmental factors [1], [2], [4], [5], [8]. In support of this, we previously reported *GADD45a* is a novel ALI/VILI candidate gene [10] that affects differential Akt ubiquitination via DNA demethylation of UCHL1, a deubiquitinating enzyme [11]. In the present study, we relied on complementary experimental approaches to characterize *GADD45a* promoter regulation by mechanical stress and to identify a functional genetic variant of *GADD45a* associated with altered mechanical stress-induced protein expression that is linked to variable susceptibility to ALI clinically.

The crucial role of GADD45a is strengthened in the current study by evidence of a gene dose effect on the elaboration of lung injury in VILI-challenged *GADD45a* ^−/−^ and *GADD45a* ^+/−^ mice. In addition, we found comparable increases in human EC *GADD45a* promoter activity in response to cyclic stretch and GADD45a mRNA levels in VILI-challenged WT mice. However, it is important to note that murine and human *GADD45a* promoters are different in sequence (alignment score 66.7) and thus their regulation by mechanical stress is likely to differ in some respects.

Our combined approach of using *GADD45a* promoter deletion constructs and ALI-associated promoter SNPs to characterize the CS-dependent regulatory elements identified two transcription factors, SP1 and IRF7, involved in *GADD45a* expression. Of note, SP1 is a transcription factor expressed in lungs that itself is upregulated in response to cyclic stretch [26] while IRF7 is known to be upregulated in the lungs of mice in response to ALI/VILI [27]. The fact that both of these transcription factors are known to be regulated in contexts relevant to ALI/VILI is particularly intriguing and helps to validate our findings.

The notion that ALI susceptibility and severity may be significantly affected by genetic factors is now well recognized. Among the earliest reports in support of this idea are associations between ALI incidence and specific polymorphisms in the genes encoding surfactant protein B [28] and angiotensin converting enzyme [29]. To date, at least 27 ALI candidate genes have been reported [30] and our group has identified several ALI-associated candidate genes including Type 2 deiodinase [5], MIF [4], myosin light chain kinase (*MYLK*) [8], pre-B-cell colony-enhancing factor (PBEF), also known as visfatin or nicotinamide phosphoribosyltransferase (NAMPT), [31] and *GADD45a* [10]. Both *PBEF* and *GADD45a* were identified via orthologous gene expression profiling of *in vivo* models of VILI and *in vitro* models of increased mechanical stress applied to human lung endothelial cells [9]. Although the function of PBEF is not precisely characterized, *PBEF* up-regulation was previously reported in human amniotic epithelial cells exposed to mechanical stress [32] and in VILI murine models [33]. Further validation for *PBEF* as a candidate gene in ALI/VILI was provided by evidence of an association of specific *PBEF* SNPs with ALI susceptibility and severity [30], [31]. Separately, the up-regulation of GADD45a has been independently verified in murine ALI/VILI [27]. These reports serve to support our candidate gene approach in general and strengthen the case for a functionally important role for GADD45a in ALI/VILI.

We utilized a tagging SNP (tSNP) approach after gene resequencing to study the association of *GADD45a* gene with sepsis/ALI. These findings identified 31 variations (29 single-base changes and 2 indels), including two variants not previously reported, and identified a significant association of *GADD45a* promoter SNP rs581000 with reduced ALI susceptibility in AA and Spanish subjects by assuming a dominant model. However, it is unclear whether the observed association is due to a specific SNP or other tSNPs or variants in nearby regions flanking the gene. Of note, SNP rs581000 exhibited LD with intergenic SNP rs607375 in the Spanish and EA study samples which was not observed in the AA study samples. To minimize potential discrepancies in our gene-association study, ancestry informative markers were previously assessed in all three cohorts (EA, AA and Spanish) [5], [6]. In addition, to account for potential bias due to population stratification, the association studies were performed in EA, AA and Spanish individuals separately. Genotyping of *GADD45a* SNPs in ALI patients revealed significant racial differences in the AA and EA from the Chicago samples. Specifically, rs581000 was associated with ALI in AAs although this was not the case for EAs, a difference supporting the increasing recognition of significant racial disparities in ALI mortality rates [34]. The genotype distribution in this study was consistent with assumptions of Hardy-Weinberg equilibrium, with exception of Spanish controls. Racial ancestry has been reported to affect the frequency of genetic variants due to excess of homozygotes because of inbreeding [35]. However, the minor allele frequency of SNP rs581000 in Spanish controls (0.28) was similar to that in a Hispanic reference panel (0.24) from dbSNP [23].

We found that allelic variation associated with the rs581000 SNP conferred both variable binding of IRF7 to this region of the *GADD45a* promoter as well as variable *GADD45a* promoter activity in CS-exposed EC. Maximum reporter activity was observed with the full-length *GADD45a* promoter fragment carrying the C allele at locus −589, suggesting IRF7 binding overrides transcription repression associated with the region −571 to −371 resulting in the upregulation of *GADD45a* in response to mechanical stress (Figure 4C). Deletion of either the IRF7 or SP1 binding regions from the *GADD45a* promoter significantly reduced the promoter activity in response to CS suggesting loss of *cis*-regulatory regions, MSRE or cooperative transcription factor complexes. However, the negative effects on transcription associated with the region −571 to −371 may indicate the presence of binding sites for specific transcription inhibitors, epigenetic modifiers or silencers in this region (Figure 4C) [13]. These results highlight the prominent functional role of *GADD45a* promoter SNP rs581000. However, it should be noted that the SNP effect observed cannot completely explain the magnitude of increase observed in promoter activity and it is possible that other SNPs also contribute to *GADD45a* promoter regulation in response to mechanical stress. To this point, two other SNPs, rs3783456 and rs3783457, located within the same region of SNP rs5810000 were identified by gene resequencing. As rs581000 is a tagging SNP the LD between these SNPs must be high and thus may also be associated with mechanical stress-mediated *GADD45a* expression and with ALI susceptibility clinically. We were not able to confirm this given the low frequency of these SNPs (Table 1). Accordingly, a full characterization of SNPs associated with GADD45a promoter activity in response to mechanical stress is an important area of further investigation.

In summary, we have identified a strong functional link between *GADD45a* expression levels and ALI susceptibility and have begun to detail specific genetic variants as well as the mechanisms underlying these observations [11]. It is imperative that our study be subjected to replication for further confirmation of our findings. We speculate that these investigations will yield further insights into ALI/VILI pathogenesis and may ultimately lead to novel therapeutic targets related to GADD45a regulation and signaling.

## Supporting Information

Table S1 Primer sequences for *GADD45a* 5′ promoter deletion constructs.
(DOCX)
