# Supplementary material for: GADD45a Promoter Regulation by a Functional Genetic Variant Associated with Acute Lung Injury
Source: PLoS One. 2014 Jun 18;9(6):e100169. doi: 10.1371/journal.pone.0100169 (PMC4062486; doi:10.1371/journal.pone.0100169)
Supplement: Table S1 — Primer sequences for GADD45a 5′ promoter deletion constructs. (DOCX) [file pone.0100169.s001.docx]

**Supplementary Table 1. Primer sequences for *GADD45a* 5ˊ promoter deletion constructs**

| Location | Forward primer | Reverse Primer | PCR (bp) |
| --- | --- | --- | --- |
| -571 | GCTTTCCAAAAATAAATCAAACCA | GCACTCACTCACAGGCGCCGCT | 806 |
| -371 | CCACCCCTTAAAACAAAAGACA | GCACTCACTCACAGGCGCCGCT | 606 |
| -133 | TTTCCGCTCCTCTCAACCT | GCACTCACTCACAGGCGCCGCT | 368 |
| +63 | TAGTGTCGTGCGGCCC | GCACTCACTCACAGGCGCCGCT | 172 |

_Recognition sites for MluI ACGCGT and Bglll AGATCT were incorporated at 5’ of forward and reverse primers respectively for molecular cloning. Reverse primer starts from position +214._
